# A phase I study of TRK-950, an Anti-CAPRIN-1 antibody, as monotherapy and in combination with nivolumab in Japanese patients with advanced solid tumors

**DOI:** 10.1007/s10637-026-01609-z

**Published:** 2026-04-14

**Authors:** Takafumi Koyama, Kan Yonemori, Jun Sato, Yuki Katsuya, Mao Okada, Tatsuya Yoshida, Fumiyoshi Okano, Noboru Yamamoto

**Affiliations:** 1 Department of Experimental Therapeutics, National Cancer Center Hospital, Tokyo, Japan; 2 Toray Industries, Inc., New Frontiers Research Laboratories and New Projects Development Division, Kamakura, Kanagawa Japan

**Keywords:** CAPRIN-1, Nivolumab, TRK-950, Japanese patients

## Abstract

CAPRIN-1 is a cytoplasmic phosphoprotein strongly expressed on the membrane surface of cancer cells in most solid tumors but not on normal cells. TRK-950 is a first-in-class humanized antibody that targets CAPRIN-1 and demonstrates antitumor effects via immune cell engagement. This phase I study assessed the tolerability and safety of TRK-950 in Japanese patients, both as monotherapy and in combination with nivolumab, and evaluated pharmacokinetic and exploratory efficacy. Part 1 involved TRK-950 monotherapy at 5 or 10 mg/kg weekly for patients with advanced solid tumors; Part 2 included combination therapy with TRK-950 (10 mg/kg weekly or 20 mg/kg biweekly) plus nivolumab (240 mg biweekly). The primary endpoints were safety and tolerability; the secondary endpoints were pharmacokinetics, immunogenicity, and exploratory antitumor activity. Thirteen patients were enrolled. No dose-limiting toxicity occurred. Adverse events (AEs) of any grade with a frequency ≥ 20% were, in Part 1, alanine aminotransferase increased, constipation, and nausea and, in Part 2, pyrexia. The incidence of AEs did not appear to be dose dependent. In Part 1, 1 patient with melanoma achieved a partial response, and the response was sustained for an extended period. In Part 2, no responses were observed. TRK-950, as monotherapy or combined with nivolumab, was safe and well tolerated in Japanese patients with observed antitumor activity. TRK-950 is currently being evaluated in ongoing clinical studies: phase II study in patients with melanoma (NCT05423262) and phase II study in patients with gastric cancer (NCT06038578).

**Trial registration** ClinicalTrials.gov, NCT05423262.

**Supplementary Information:**

The online version contains supplementary material available at 10.1007/s10637-026-01609-z.

## Introduction

Cytoplasmic activation/proliferation-associated protein-1 (CAPRIN-1) is a highly conserved phosphoprotein that is present in the cytoplasm [1–3]. The expression of CAPRIN-1 is induced during cell activation from a resting state and during cell division and it is required for cell proliferation [1, 4]. In addition, because a portion of CAPRIN-1 is strongly expressed on the membrane surface of cancer cells in most solid tumors but is not expressed on the surface of normal cells, it is considered a promising target for antibody therapeutics in solid tumors [5]. To treat patients with solid tumors in which CAPRIN-1 is expressed on the cancer cell membrane surface, the humanized anti-CAPRIN-1 monoclonal immunoglobulin G subclass 1 (IgG1) antibody “TRK-950” was developed by TORAY Industries, Inc. [5]. TRK-950 exerts antitumor activity against CAPRIN-1-expressing cancer cells through antibody-dependent cellular cytotoxicity (ADCC) and antibody-dependent cellular phagocytosis (ADCP) mediated by binding to immune cells such as natural killer (NK) cells and macrophages.

A phase I clinical study (NCT02990481) of TRK-950 monotherapy in patients with solid tumors has been conducted in the U.S. and France as a first-in-human study. It showed that TRK-950 has a favorable safety profile and can be safely administered. In addition, preliminary evidence of potential antitumor activity in patients with CAPRIN-1 expression in tumors was observed [6]. Thereafter, a phase Ib clinical study (NCT03872947) was conducted in patients with cancers for which existing anticancer drugs are indicated to evaluate the safety and pharmacokinetics (PK) of TRK-950 in combination therapy with each anticancer drug and to determine the recommended dose and administration method for future studies [7].

This paper reports the results of an evaluation of the safety, tolerability, PK, and preliminary antitumor activity of TRK-950, administered either as monotherapy or in combination with nivolumab, in Japanese patients with malignant solid tumors.

## Patients and methods

### Study design and objectives

This study was a single-center, open-label, phase I study (NCT05423262) conducted in Japan. It consisted of three parts, monotherapy (Part 1) for patients with advanced solid tumors; combination therapy with nivolumab (Part 2) for patients with advanced solid tumors eligible for nivolumab treatment. This paper reports the findings from Parts 1 and 2, where randomization and blinding were not applied.

The primary objective of Part 1 was to evaluate the safety and tolerability of TRK-950. TRK-950 was administered intravenously weekly at 5 mg/kg or 10 mg/kg, with each cycle lasting 28 days. The starting dose was 5 mg/kg TRK-950 weekly, and dose escalation to the next level, 10 mg/kg TRK-950 weekly, was determined on the basis of DLT assessment using a standard 3 + 3 design.

The primary objective of Part 2 was to evaluate the safety and tolerability of TRK-950 in combination with nivolumab. Each cycle lasted 28 days, with TRK-950 administered intravenously at 10 mg/kg weekly or 20 mg/kg biweekly. Nivolumab was administered intravenously at 240 mg biweekly. The starting dose was 10 mg/kg TRK-950 weekly in combination with nivolumab, and the decision on whether to proceed to the next dose level, which was 20 mg/kg TRK-950 biweekly in combination with nivolumab, was made on the basis of DLT assessment using a standard 3 + 3 design.

The study protocol and other relevant documents were approved by the relevant Institutional Review Boards, and all participants provided written informed consent.

### Patient eligibility

In Part 1, patients of either sex with histologically and cytologically confirmed locally advanced or metastatic solid tumors who were refractory or intolerant to standard therapies or for whom no standard therapy existed were enrolled. The key inclusion criteria were: age 18 years or older, life expectancy of at least 3 months, presence of target or nontarget lesions according to the Response Evaluation Criteria in Solid Tumors (RECIST) v1.1, Eastern Cooperative Oncology Group (ECOG) performance status of 0 or 1, and adequate organ function.

In Part 2, patients of either sex with histologically and cytologically confirmed locally advanced or metastatic solid tumors who were eligible for standard therapy with nivolumab monotherapy were enrolled. The key inclusion criteria were the same as those applied in Part 1.

### Assessments

#### Safety, DLTs, and MTD

AEs were assessed according to the Common Terminology Criteria for Adverse Events (CTCAE), Version 5.0 (Japanese edition). All AEs that occurred after the first administration of the study treatment and within 28 days after the last administration were recorded.

DLT was defined as the following toxicities related to TRK-950 occurring during the DLT evaluation period (within 28 days from the first administration of TRK-950):

- Grade 4 neutropenia lasting for ≥ 5 days;
- Grade 3 or 4 neutropenia with pyrexia and/or infection;
- Grade 3 thrombocytopenia with bleeding;
- Grade 4 thrombocytopenia, Grade 3 or 4 nonhematologic toxicity;
- AEs or abnormal laboratory values that resulted in withholding TRK-950 on ≥ 2 occasions;
- AEs or abnormal laboratory values that resulted in withholding nivolumab for ≥ 50% of the scheduled doses (Part 2 only).

In Part 1 and Part 2, a dose was considered tolerable if < 33% of patients experienced a DLT. Patients who received less than 75% of the planned TRK-950 doses during the DLT evaluation period were excluded from the DLT evaluation.

#### Pharmacokinetics

For the PK analysis of TRK-950 and nivolumab, serum samples were collected as described in Table S1. Serum concentrations of TRK-950 and nivolumab were measured using validated immunoassay methods. For TRK-950, serum concentrations at the first administration in cycle 1 and for nivolumab, serum concentrations at the first coadministration of TRK-950 and nivolumab in cycles 1 and 3 were analyzed using noncompartmental analysis with Phoenix WinNonlin (Version 8.4; Certara USA, Inc., 2023; RRID: SCR_024504).

#### Cytokines

In Part 1, serum concentrations of interferon-gamma (IFN-γ), interleukin (IL)-1β, IL-2, IL-6, IL-10, and tumor necrosis factor-alpha (TNF-α) were measured using an enzyme immunoassay (EIA) to evaluate potential cytokine-mediated alterations in cytochrome P450 (CYP) activity.

#### Antitumor activity

Tumor response was assessed by the investigator according to RECIST v1.1. Tumor imaging for evaluation was performed every 2 cycles during TRK-950 treatment up to cycle 6 and every 3 cycles thereafter.

#### Immunohistochemical analysis for CAPRIN-1

CAPRIN-1 expression on the tumor cell membrane was assessed by immunohistochemistry (IHC) using a rabbit anti-human CAPRIN-1 monoclonal antibody. At screening, tumor tissue obtained by biopsy from either the primary tumor or a metastatic lesion was analyzed for all patients at a central pathology laboratory. The staining intensity of CAPRIN-1 and the percentage of tumor cells exhibiting membranous staining were evaluated. Patients were considered to have tumors exhibiting membranous CAPRIN-1 immunoreactivity if staining was detected in tumor cells.

Staining intensity was scored on a four-point scale: 0 (no membrane staining), 1 + (faint membrane staining), 2 + (weak to moderate membrane staining), and 3 + (strong membrane staining).

#### Immunogenicity

Immunogenicity was assessed by the presence of anti-TRK-950 antibodies detected by a validated electrochemiluminescence (ECL)-based bridging immunoassay. Blood samples were collected prior to TRK-950 administration on day 1 of cycle 1, predosed on day 15 of cycle 1, predosed on day 1 of cycle 2 and subsequent cycles, and at the follow-up visit.

### Statistical considerations

The sample size for Part 1 was determined on the basis of a traditional 3 + 3 dose-escalation design. For the sample size of Part 2, a 3 + 3 design was also applied, although it is not a dose-escalation study. The safety population included all patients who received at least one dose of the study drug. The efficacy population included all patients who received at least one dose of the study drug and had at least one tumor assessment evaluation after administration. Descriptive statistics, including the mean, median, standard deviation, and range for continuous variables and the frequency and percentage for categorical variables, were used to summarize the data. No statistical hypothesis testing was planned. The Wilson score interval was used to calculate confidence intervals for a binomial proportion. Antitumor activity was visually summarized using waterfall plots to depict the best percentage change in target lesion size, and swimmer plots were constructed to represent treatment duration and overall response. All the statistical analyses, excluding those related to figures, were performed using SAS version 9.4 (SAS Institute, RRID: SCR_008567).

## Results

### Patient characteristics

A total of 13 patients were enrolled. As shown in Fig. S1, 7 patients were included in Part 1, and 6 patients were included in Part 2. In Part 1, 1 patient who received TRK-950 at 10 mg/kg was excluded from the DLT evaluation because, owing to an AE unrelated to TRK-950, the patient was able to receive only 2 doses during the 28-day DLT evaluation period. Safety data for this patient were included in the overall safety; To compensate for the DLT assessment that could not be conducted in this patient, an additional patient was enrolled.

The baseline characteristics of the treated patients are summarized in Table 1. The cancer organ varied among individual patients. All patients had received at least 2 prior systemic therapies, and most had a history of 4 or more prior systemic therapies.

**Table 1:** Baseline characteristics of treated patients

Characteristics	TRK-950 5 mg/kg weekly, n (%)	TRK-950 10 mg/kg weekly, n (%)	All, n (%)^a^	TRK-950 10 mg/kg weekly + NIVO, n (%)	TRK-950 20 mg/kg biweekly + NIVO, n (%)	All, n (%)^a^
Number of patients	3	4	7	3	3	6
Age (years), median (range)	52.0 (52–72)	55.0 (48–73)	52.0 (48–73)	58.0 (56–75)	68.0 (52–68)	63.0 (52–75)
Sex						
Male	1 (33.3)	2 (50)	3 (42.9)	3 (100)	2 (66.7)	5 (83.3)
Female	2 (66.7)	2 (50)	4 (57.1)	0	1 (33.3)	1 (16.7)
Performance status (ECOG)						
0	1 (33.3)	3 (75)	4 (57.1)	2 (66.7)	3 (100)	5 (83.3)
1	2 (66.7)	1 (25)	3 (42.9)	1 (33.3)	0	1 (16.7)
Primary tumor						
Colorectal cancer	0	2 (50)	2 (28.6)	0	0	0
Melanoma	0	1 (25)	1 (14.3)	1 (33.3)	0	1 (16.7)
Ovary	1 (33.3)	0	1 (14.3)	0	0	0
Pancreas	1 (33.3)	1 (25)	2 (28.6)	0	0	0
Cancer of unknown primary	1 (33.3)	0	1 (14.3)	0	0	0
Esophagus	0	0	0	1 (33.3)	0	1 (16.7)
Kidney	0	0	0	0	1 (33.3)	1 (16.7)
Lung	0	0	0	1 (33.3)	1 (33.3)	2 (33.3)
Oropharyngeal	0	0	0	0	1 (33.3)	1 (16.7)
Histology						
Adenocarcinoma	1 (33.3)	3 (75)	4 (57.1)	0	1 (33.3)	1 (16.7)
Squamous cell carcinoma	0	0	0	2 (66.7)	1 (33.3)	3 (50)
Other	2 (66.7)	1 (25)	3 (42.9)	1 (33.3)	1 (33.3)	2 (33.3)
Prior systemic therapy (lines)						
≤ 3	1 (33.3)	1 (25)	2 (28.6)	1 (33.3)	0	1 (16.7)
≥ 4	2 (66.7)	3 (75)	5 (71.4)	2 (66.7)	3 (100)	5 (83.3)
Screening CAPRIN-1 expression^b^						
Yes	3 (100)	4 (100)	7 (100)	3 (100)	3 (100)	6 (100)
No	0	0	0	0	0	0

*NIVO* nivolumab, *ECOG* eastern cooperative oncology group

^a^ Patients who received one or more dose of TRK-950

^b^ Among patients who received the treatment, the staining intensity of CAPRIN‑1 and the percentage of tumor cells exhibiting membranous staining were as follows: Pt#1, 3 +/50%; Pt#2, 1 +/20%; Pt#3, 3 +/10%; Pt#4, 1 +/10% >; Pt#5, 1 +/10% >; Pt#6, 1 +/20%; Pt#7, 1 +/10% >; Pt#8, 2 +/30%; Pt#9, 1 +/10% >; Pt#10, 1 +/10% >; Pt#11, 1 +/30%; Pt#12, 1 +/10% >; and Pt#13, 2 +/10% <

In Part 1, the median patient age was 52 years (range, 48–73 years); 3 patients (42.9%) had an ECOG performance status of 1, and 4 patients (57.1%) had a status of 0.

In Part 2, the median patient age was 63 years (range, 52–75 years); 1 patient (16.7%) had an ECOG performance status of 1, and 5 patients (83.3%) had a status of 0. All patients had a history of immunotherapy; all 6 patients had a history of treatment with PD-1 inhibitors, and in 2 of these patients, combination therapy with a CTLA-4 inhibitor was administered.

### Safety

All treatment-emergent adverse events (TEAEs) from Parts 1 and 2, as well as treatment-related adverse events (TRAEs) considered to be associated with TRK-950, are presented in Table 2.

**Table 2:** The incidence of adverse events

MedDRA preferred term	All AEs						Grade 3 or higher AEs					
	TRK-950 5 mg/kg weekly, n	TRK-950 10 mg/kg weekly, n	All, *n* (%)	TRK-950 10 mg/kg weekly + NIVO, n	TRK-950 20 mg/kg biweekly + NIVO, n	All, *n* (%)	TRK-950 5 mg/kg weekly, n	TRK-950 10 mg/kg weekly, n	All, *n* (%)	TRK-950 10 mg/kg weekly + NIVO, n	TRK-950 20 mg/kg biweekly + NIVO, n	All, *n* (%)
Number of patients	3	4	7	3	3	6	3	4	7	3	3	6
**Any TEAE**	3	4	7 (100)	3	3	6 (100)	1	3	4 (57.1)	1	1	2 (33.3)
Alanine aminotransferase increased	0	2	2 (28.6)	0	0	0 (0)	0	0	0 (0)	0	0	0 (0)
Constipation	0	2	2 (28.6)	0	0	0 (0)	0	0	0 (0)	0	0	0 (0)
Nausea	2	0	2 (28.6)	0	0	0 (0)	0	0	0 (0)	0	0	0 (0)
Abdominal pain lower	1	0	1 (14.3)	0	0	0 (0)	0	0	0 (0)	0	0	0 (0)
Anaphylactic reaction	0	1	1 (14.3)	0	0	0 (0)	0	1	1 (14.3)	0	0	0 (0)
Anxiety	0	1	1 (14.3)	0	0	0 (0)	0	0	0 (0)	0	0	0 (0)
Aspartate aminotransferase increased	0	1	1 (14.3)	0	0	0 (0)	0	0	0 (0)	0	0	0 (0)
Atrial fibrillation	0	1	1 (14.3)	0	0	0 (0)	0	0	0 (0)	0	0	0 (0)
Blood alkaline phosphatase increased	0	1	1 (14.3)	0	0	0 (0)	0	0	0 (0)	0	0	0 (0)
Blood creatinine increased	1	0	1 (14.3)	0	0	0 (0)	0	0	0 (0)	0	0	0 (0)
Decreased appetite	1	0	1 (14.3)	0	0	0 (0)	0	0	0 (0)	0	0	0 (0)
Diarrhea	1	0	1 (14.3)	0	0	0 (0)	0	0	0 (0)	0	0	0 (0)
Dry eye	0	1	1 (14.3)	0	0	0 (0)	0	0	0 (0)	0	0	0 (0)
Electrocardiogram QT prolonged	1	0	1 (14.3)	0	0	0 (0)	1	0	1 (14.3)	0	0	0 (0)
Gastritis	0	1	1 (14.3)	0	0	0 (0)	0	0	0 (0)	0	0	0 (0)
Headache	1	0	1 (14.3)	0	0	0 (0)	0	0	0 (0)	0	0	0 (0)
Hypertension	1	0	1 (14.3)	0	0	0 (0)	0	0	0 (0)	0	0	0 (0)
Infusion related reaction	1	0	1 (14.3)	0	0	0 (0)	0	0	0 (0)	0	0	0 (0)
Lipase increased	1	0	1 (14.3)	0	0	0 (0)	0	0	0 (0)	0	0	0 (0)
Malaise	1	0	1 (14.3)	0	1	1 (16.7)	0	0	0 (0)	0	1	1 (16.7)
Pancreatitis acute	0	1	1 (14.3)	0	0	0 (0)	0	1	1 (14.3)	0	0	0 (0)
Platelet count decreased	0	1	1 (14.3)	0	0	0 (0)	0	0	0 (0)	0	0	0 (0)
Pneumonia	0	1	1 (14.3)	0	0	0 (0)	0	1	1 (14.3)	0	0	0 (0)
Rash	1	0	1 (14.3)	1	0	1 (16.7)	0	0	0 (0)	0	0	0 (0)
Seizure	0	1	1 (14.3)	0	0	0 (0)	0	1	1 (14.3)	0	0	0 (0)
Urticaria	0	1	1 (14.3)	0	0	0 (0)	0	0	0 (0)	0	0	0 (0)
Vomiting	1	0	1 (14.3)	1	0	1 (16.7)	0	0	0 (0)	0	0	0 (0)
Weight decreased	1	0	1 (14.3)	0	0	0 (0)	0	0	0 (0)	0	0	0 (0)
Anemia	0	0	0 (0)	1	0	1 (16.7)	0	0	0 (0)	0	0	0 (0)
Arthralgia	0	0	0 (0)	1	0	1 (16.7)	0	0	0 (0)	0	0	0 (0)
Bronchitis	0	0	0 (0)	1	0	1 (16.7)	0	0	0 (0)	0	0	0 (0)
Cough	0	0	0 (0)	0	1	1 (16.7)	0	0	0 (0)	0	0	0 (0)
Dry skin	0	0	0 (0)	1	0	1 (16.7)	0	0	0 (0)	0	0	0 (0)
Eye discharge	0	0	0 (0)	0	1	1 (16.7)	0	0	0 (0)	0	0	0 (0)
Fatigue	0	0	0 (0)	1	0	1 (16.7)	0	0	0 (0)	1	0	1 (16.7)
Gastroesophageal reflux disease	0	0	0 (0)	0	1	1 (16.7)	0	0	0 (0)	0	0	0 (0)
Hematuria	0	0	0 (0)	0	1	1 (16.7)	0	0	0 (0)	0	1	1 (16.7)
Pyrexia	0	0	0 (0)	3	0	3 (50.0)	0	0	0 (0)	0	0	0 (0)
Scrotal edema	0	0	0 (0)	1	0	1 (16.7)	0	0	0 (0)	0	0	0 (0)
**Any TRAE**	2	1	3 (42.9)	2	0	2 (33.3)	1	0	1 (14.3)	0	0	0 (0)
Alanine aminotransferase increased	0	1	1 (14.3)	0	0	0 (0)	0	0	0 (0)	0	0	0 (0)
Aspartate aminotransferase increased	0	1	1 (14.3)	0	0	0 (0)	0	0	0 (0)	0	0	0 (0)
Blood alkaline phosphatase increased	0	1	1 (14.3)	0	0	0 (0)	0	0	0 (0)	0	0	0 (0)
Electrocardiogram QT prolonged	1	0	1 (14.3)	0	0	0 (0)	1	0	1 (14.3)	0	0	0 (0)
Headache	1	0	1 (14.3)	0	0	0 (0)	0	0	0 (0)	0	0	0 (0)
Infusion related reaction	1	0	1 (14.3)	0	0	0 (0)	0	0	0 (0)	0	0	0 (0)
Malaise	1	0	1 (14.3)	0	0	0 (0)	0	0	0 (0)	0	0	0 (0)
Nausea	1	0	1 (14.3)	0	0	0 (0)	0	0	0 (0)	0	0	0 (0)
Platelet count decreased	0	1	1 (14.3)	0	0	0 (0)	0	0	0 (0)	0	0	0 (0)
Vomiting	1	0	1 (14.3)	1	0	1 (16.7)	0	0	0 (0)	0	0	0 (0)
Weight decreased	1	0	1 (14.3)	0	0	0 (0)	0	0	0 (0)	0	0	0 (0)
Pyrexia	0	0	0 (0)	2	0	2 (33.3)	0	0	0 (0)	0	0	0 (0)

*AE* adverse event, *NIVO* nivolumab, *TRAE* treatment related adverse event

In Part 1, no DLTs were observed, and the MTD could not be determined. All 7 patients (100.0%) experienced at least 1 TEAE, and TRAEs were observed in 3 patients (42.9%). AEs that occurred in > 20% or more of the patients were alanine aminotransferase increased (*n* = 2, 28.6%), constipation (*n* = 2, 28.6%), and nausea (*n* = 2, 28.6%). Grade 3 or higher AEs were observed in 4 patients (57.1%). These included an anaphylactic reaction (*n* = 1, received 10 mg/kg), electrocardiogram QT prolonged (*n* = 1, received 5 mg/kg), pancreatitis acute (*n* = 1, received 10 mg/kg), pneumonia (*n* = 1, received 10 mg/kg), and seizure (*n* = 1, received 10 mg/kg). Among these, only the electrocardiogram QT prolonged was determined to be related to TRK-950.

In Part 2, no DLTs were observed, and both 10 mg/kg weekly TRK-950 and 20 mg/kg biweekly TRK-950 were tolerated. All 6 patients (100.0%) experienced at least 1 TEAE, and TRAEs related to TRK-950 were observed in 2 patients (33.3%). The only AE observed in > 20% or more of the patients was pyrexia (*n* = 3, 50.0%). Grade 3 or higher AEs were observed in 2 patients (33.3%). These included malaise (*n* = 1; received 20 mg/kg TRK-950 biweekly), fatigue (*n* = 1; received 10 mg/kg TRK-950 weekly), and hematuria (*n* = 1; received 20 mg/kg TRK-950 biweekly). All of these events were determined to be unrelated to TRK-950.

Across Parts 1 and Part 2, no treatment discontinuations due to AEs or Grade 5 AEs were reported. Immune-related adverse events (irAEs) associated with TRK-950 were observed only in Part 1, with three events reported in a single patient: aspartate aminotransferase increased (Grade 2), alanine aminotransferase increased (Grade 1), and blood alkaline phosphatase increased (Grade 1). In addition, irAEs associated with nivolumab were observed in Part 2, with one event reported in a single patient, rash (Grade 1).

### Pharmacokinetics

For Parts 1 and 2, the PK parameters at the first administration of TRK-950 (Cycle 1) are summarized in Table 3, and the time course of serum TRK-950 concentrations is shown in Fig. S2.

**Table 3:** Pharmacokinetic parameters of TRK-950 after the first dose (Cycle 1; noncompartmental analysis)

Parameter	TRK-950 5 mg/kg weekly	TRK-950 10 mg/kg weekly	TRK-950 10 mg/kg weekly + NIVO	TRK-950 20 mg/kg biweekly + NIVO
Number of patients	3	4	3	3
C_max_ (μg/mL)^a^	97.0 ± 4.4	181 ± 55	264 ± 77	506 ± 89
T_max_ (h)^b^	1.0 (1.0–6.8)	1.0 (1.0–2.0)	1.2 (1.2–7.1)	1.1 (1.0–24.5)
AUC_last_ (μg⸱h/mL)^a^	9860 ± 790	14800 ± 4000	20700 ± 5900	62900 ± 4100
AUC_inf_ (μg⸱h/mL)^a^	21200 ± 7000	27600 ± 8000	32400 ± 6000	85400 ± 9900
C_avg_ (μg/mL)^a^	55.8 ± 7.1	101 ± 28	129 ± 34	186 ± 12
C_min_ (μg/mL)^a^	40.1 ± 10.2	67.0 ± 16.9	75.8 ± 10.6	84.2 ± 29.7
CL (mL/h/kg)^a^	0.254 ± 0.082	0.385 ± 0.108	0.317 ± 0.064	0.236 ± 0.028
V_ss_ (mL/kg)^a^	63.3 ± 8.4	72.6 ± 20.2	50.3 ± 16.4	57.5 ± 14.9
T_1/2_ (day)^b^	6.1 (5.6–11.6)	5.4 (4.9–6.2)	4.7 (3.8–4.8)	7.5 (4.6–9.9)

*AUC*_*inf*_ Area under the serum concentration–time curve from time zero extrapolated to infinity, *AUC*_*last*_ Area under the serum concentration–time curve from time zero to the last quantifiable time point, *C*_*avg*_ Average serum concentration (AUC_last_ divided by the last quantifiable time point), *C*_*max*_ maximum serum concentration, *C*_*min*_ trough serum concentration, *CL* systemic clearance, *T*_*max*_ time to reach maximum serum concentration, *T*_*1/2*_ elimination half-life, *V*_*ss*_ apparent volume of distribution at steady state, *NIVO* nivolumab

^a^ Mean (standard deviation) is presented for C_max_, AUC_last_, AUC_inf_, C_avg_, C_min_, CL and V_ss_

^b^ Median (minimum, maximum) is presented for T_max_ and T_1/2_

In Part 1, when TRK-950 was administered as monotherapy, the systemic clearance (CL), apparent volume of distribution at steady state (V_ss_), and elimination half-life (T_1/2_) ranged from 0.254–0.385 mL/h/kg, 63.3–72.6 mL/kg, and 5.4–6.1 days, respectively.

In Part 2, when TRK-950 was administered in combination with nivolumab, the CL, V_ss_, and T_1/2_ of TRK-950 ranged from 0.236–0.317 mL/h/kg, 50.3–57.5 mL/kg, and 4.7–7.5 days, respectively. For nivolumab, the maximum serum concentration (C_max_), trough serum concentration (C_min_), and average serum concentration (C_avg_) were 80.1, 21.7, and 34.6 μg/mL, respectively (Table S2).

### Antitumor activity

**Fig. 1:**
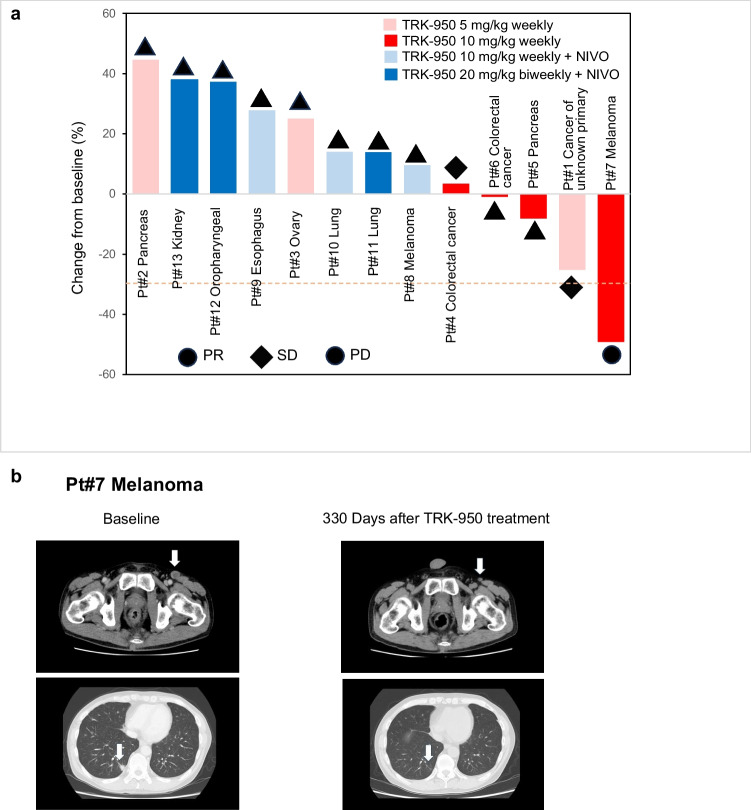

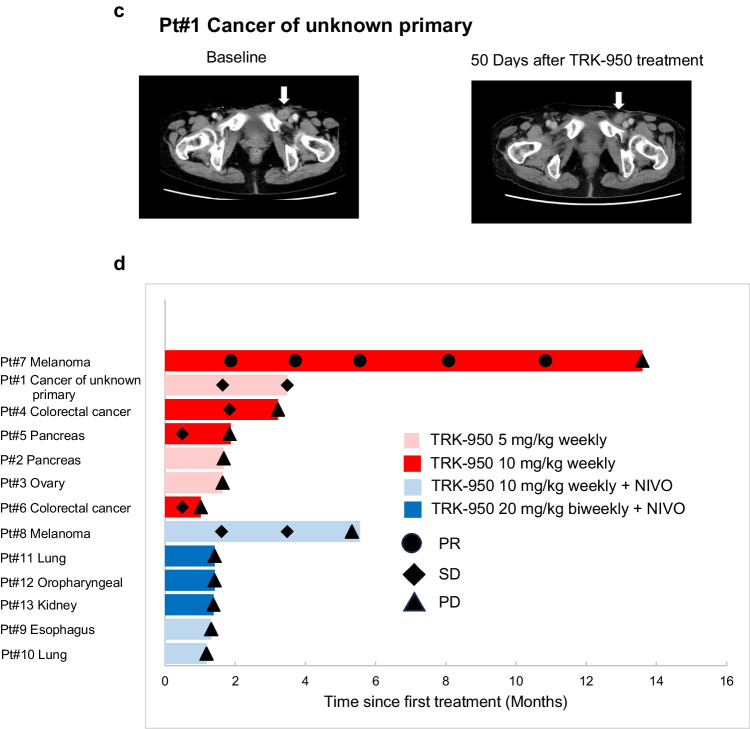
Treatment response in patients (**a**) The best percentage change from baseline in the sum of the largest diameters of measurable tumors in 13 patients for whom data were available from both baseline and postbaseline assessments of target lesions. The lower dashed line indicates a 30% decrease in tumor size. Abbreviations: PR, partial response; SD, stable disease; PD, progressive disease; Pt, patient. (**b**) Representative antitumor activity in patients with melanoma. CT scans of the metastatic lung and inguinal lymph node lesion. (**c**) Representative antitumor activity in patients with CT scans of the metastatic inguinal lymph node lesion. (**d**) Swimmer plot showing the treatment durations for all 13 activity-evaluable patients. Duration of treatment and response with TRK-950. Each bar represents 1 patient. The length of the bar represents the duration from the first administration of the study drug to the date of discontinuation of treatment. Abbreviations: PR, partial response; SD, stable disease; PD, progressive disease; Pt, patient

The best overall response (BOR) and disease control rate (DCR) in Parts 1 and 2 are presented in Table 4. The percentage change in target lesions from baseline is shown in Fig. 1a.

**Table 4:** Summary of best overall response and disease control rate

Variables	TRK-950 5 mg/kg weekly, *n* (%)	TRK-950 10 mg/kg weekly, *n* (%)	All, *n* (%)	TRK-950 10 mg/kg weekly + NIVO, *n* (%)	TRK-950 20 mg/kg biweekly + NIVO, *n* (%)	All, *n* (%)
Number of patients^a^	3	4	7	3	3	6
Best overall response categories						
Complete response	0	0	0	0	0	0
Partial response	0	1 (25)	1 (14.3)	0	0	0
Stable disease	1 (33.3)	1 (25)	2 (28.6)	1 (33.3)	0	1 (16.7)
Progressive disease	2 (66.7)	2 (50)	4 (57.1)	2 (66.7)	3 (100)	5 (83.3)
Not evaluable	0	0	0	0	0	0
Disease control rate^b^						
n (%)	1 (33.3)	2 (50)	3 (42.9)	1 (33.3)	0	1 (16.7)
95% CI	(6.1–79.2)	(15.0–85.0)	(15.8–75.0)	(6.1–79.2)	(0.0–56.1)	(3.0–56.4)
Overall response rate						
n (%)	0	1 (25)	1 (14.3)	0	0	0
95% CI^c^	(0.0–56.1)	(4.6–66.9)	(2.6–51.3)	(0.0–56.1)	(0.0–56.1)	(0.0–39.0)

*NIVO* nivolumab, *CI* confidence interval

^a^ N represents the number of patients assigned to the respective dosage and administration and is the denominator for percentages unless otherwise indicated. Response is based on investigator assessment using RECIST 1.1

^b^ Disease control rate is defined as the proportion of patients who achieved a complete response, partial response, or stable disease as their best overall response in accordance with RECIST 1.1 criteria

^c^ CIs were calculated using the Wilson score method

In Part 1, 1 patient with melanoma achieved a partial response (PR), resulting in an overall response (ORR) of 14.3%. This patient had previously received nivolumab as an adjuvant therapy and, after recurrence with distant metastases, was treated with a combination of nivolumab and ipilimumab. The administration of 10 mg/kg TRK-950 was initiated 50 days after the last treatment with nivolumab and ipilimumab. After starting TRK-950 treatment, the size of the target lesions (left inguinal lymph node and lung) was reduced by 38.4% at 8 weeks, with a maximum reduction of 49.2% achieved (Fig. 1b). TRK-950 treatment was continued for 407 days, and this PR was maintained for approximately 1 year (Fig. 1d). In addition, 1 patient with cancer of unknown primary status who received 5 mg/kg TRK-950 and 1 patient with colorectal cancer who received 10 mg/kg TRK-950 achieved stable disease (SD), resulting in a DCR of 42.9%. A patient with cancer of unknown primary status also experienced a certain degree of tumor shrinkage, with a maximum reduction of 25.2%. This patient had received four prior lines of therapy, including chemotherapy and nivolumab. The administration of 5 mg/kg TRK-950 was initiated 125 days after the last treatment with nivolumab. After starting TRK-950 treatment, the size of the target lesion (left inguinal lymph node) was reduced by 24.2% at 8 weeks (Fig. 1c).

In Part 2, none of the 6 patients achieved a response. One patient with melanoma who received 10 mg/kg TRK-950 weekly in combination with nivolumab achieved a SD, resulting in a DCR of 16.7%.

### Cytokines

In Part 1, transient and mild increases in serum IL-6, TNF-α, and IFN-γ concentrations were observed following TRK-950 administration. Values generally trended back toward baseline by the Day 8 pre-dose time point, although residual elevations persisted in some participants (e.g., one participant with pancreatic cancer receiving 10 mg/kg weekly) and returned toward baseline by the Day 15 or Day 22 pre-dose time points. No changes were observed in IL-1β or IL-10 after dosing, and IL-2 concentrations were below the lower limit of quantification (31.3 pg/mL) in all samples (Fig. S3).

### Immunogenicity

In Part 1, anti-TRK-950 antibodies were not detected in any of the 7 patients who received TRK-950 monotherapy. In Part 2, among the 6 patients who received combination therapy with nivolumab, 1 patient with melanoma who was administered TRK-950 10 mg/kg weekly in combination with nivolumab tested positive for anti-TRK-950 antibodies; further evaluation for the presence of neutralizing antibodies (Nabs) revealed a positive result. However, in this case, both anti-TRK-950 antibodies and NAbs were already positive before the administration of TKR-950, and both became negative after treatment was continued (Tables S3 and S4).

## Discussion

In Part 1 of this phase I study, the planned monotherapy dosages for Japanese cancer patients were 5 mg/kg weekly and 10 mg/kg weekly. Preclinically, TRK-950 administered at 10 mg/kg weekly showed the greatest antitumor effect, with a bell-shaped dose–response, and efficacy decreased above this level in tumor-bearing mouse models [5]. In a phase I clinical study conducted in the U.S. and France, TRK-950 monotherapy was escalated to 30 mg/kg weekly without DLTs, confirming tolerability [6]. On the basis of the PK profile from this phase I clinical study and pharmacokinetic/pharmacodynamic analyses using nonclinical data [5], the effective clinical dosage range of TRK-950 was predicted to start at 5 mg/kg weekly and peak at 10 mg/kg weekly. Accordingly, this phase I study conducted in Japan set 5 mg/kg weekly as the starting dosage and 10 mg/kg weekly as the maximum dosage, which is considered the optimal dosage. The tolerability of TRK-950 monotherapy in Japanese cancer patients was evaluated within this dosing range.

TRK-950 monotherapy had no DLTs at either dosage. No clinically meaningful dosage-dependent trends in AEs were observed, which is consistent with the results of the phase I study conducted in the U.S. and France [6].

With respect to efficacy, a durable PR was achieved in one patient with melanoma at 10 mg/kg weekly, which was considered the optimal dosage for TRK-950. In addition, tumor shrinkage (-25.2%) was also observed in a patient with cancer of unknown primary at 5 mg/kg weekly, which was considered the threshold dosage for initiating therapeutic effects.

In this phase I study, Part 1 was conducted under the assumption that 10 mg/kg weekly would be the optimal dosage for TRK-950 monotherapy. The results, in terms of both safety and efficacy, did not contradict this assumption, providing a rationale for further development at 10 mg/kg weekly.

The PK profile of TRK-950 monotherapy was similar to that of a typical IgG1 antibody [8–10]. The PK parameters of Japanese cancer patients did not markedly differ from those of non-Japanese patients in the phase I clinical study conducted jointly in the U.S. and France, suggesting that the PK parameters of TRK-950 do not differ across ethnicities.

TRK-950 is not designed to directly regulate cytokines, and no substantial or sustained changes in serum cytokine levels were observed following TRK-950 monotherapy. Although CYP activity or expression was not directly assessed, the absence of sustained cytokine elevations suggests a low likelihood of clinically meaningful cytokine-mediated modulation of CYP enzymes. Accordingly, cytokine-mediated CYP-related drug–drug interactions with small-molecule drugs are considered unlikely under the conditions evaluated.

In Part 2, Japanese cancer patients were treated with TRK-950 (10 mg/kg weekly or 20 mg/kg biweekly) plus nivolumab. The mechanisms of action of TRK-950 include ADCP activity mediated by macrophages and ADCC activity mediated by natural killer (NK) cells. On the other hand, it has been reported that programmed death-1 (PD-1) is also expressed on immune cells within tumors and that immune checkpoint inhibitors such as nivolumab can activate the effector functions of these immune cells [11–14]. Therefore, nivolumab might enhance the efficacy of TRK-950.

For the TRK-950 dosage, 10 mg/kg weekly was selected on the basis of the results from Part 1 of this Phase I study. In addition, based on PK data from the prior first-in-human Phase I study [6], 20 mg/kg biweekly was expected to provide broadly comparable exposure to 10 mg/kg weekly. Assuming similar clinical potency, the 20 mg/kg biweekly dosage, which offers greater convenience, was also included.

The transition from 10 mg/kg weekly TRK-950 to 20 mg/kg biweekly was completed without DLTs, and no significant differences in AE incidence/severity were observed between the 10 mg/kg weekly and 20 mg/kg biweekly regimens. The safety profile was favorable and comparable to that of Part 1, with no worsening, and there was no trend toward worsening AEs known to be associated with nivolumab.

To assess PK interactions, the effects of TRK-950 at 10 mg/kg weekly plus nivolumab were compared with those of TRK-950 monotherapy. Combination therapy resulted in higher serum concentrations of TRK-950, yet it was within the variability observed in the phase I clinical study conducted in the U.S. and France. Mechanistically, nivolumab is unlikely to directly alter the PK of TRK-950, and interindividual variability is the more plausible explanation. Serum exposures (C_avg_, C_min_, C_max_) of nivolumab were within the 95% prediction interval of nivolumab exposure estimated by a population PK model based on clinical study data [15]. Thus, the combination did not affect the PK profile of either TRK-950 or nivolumab.

Serum exposure metrics for TRK-950 indicated higher peak concentrations (C_max_) with 20 mg/kg biweekly dosing, as expected from the higher administered dose, while trough concentrations (C_min_) were similar to those observed with 10 mg/kg weekly dosing. Interval-averaged exposure (C_avg_) was modestly higher with 20 mg/kg biweekly. Together with the similar safety profile observed in this dataset, these findings support consideration of a more convenient biweekly dosing regimen for further evaluation.

In Part 2, no responses were observed, and the DCR was 16.7%, reflecting limited activity in a small, heavily pretreated cohort (prior therapies ranged from 2 to 8). All patients had prior PD-1 inhibitor treatment, representing a rechallenge setting. Future studies should evaluate combination therapy in larger, more homogeneous populations.

Immunogenicity was evaluated in 13 patients across Parts 1 and 2. Anti-TRK-950 antibodies, with positive NAbs, were detected in one patient with melanoma receiving TRK-950 plus nivolumab. We employed a newly developed ADA assay method that combines Melon™ gel with SPEAD pretreatment, which we developed to remove nonantibody proteins that can cause interindividual variability and drug interference [16]. Despite the application of this method, both anti-TRK-950 antibodies and NAbs were present before TRK-950 was administered and became negative during TRK-950 treatment. Therefore, the anti-TRK-950 antibodies observed in this case are considered to be immunoglobulin molecules that were already present before administration, rather than anti-TRK-950 antibodies induced by TRK-950 treatment, and were considered a phenomenon independent of TRK-950 treatment. Furthermore, the anti-TRK-950 antibodies detected in this patient had no effect on the trough blood concentration of TRK-950, indicating that the immunogenicity risk for TRK-950 is negligible.

In summary, TRK-950 was well tolerated in patients with advanced solid tumors, both as monotherapy and in combination with nivolumab, and demonstrated clinical utility. TRK-950 is currently being evaluated in ongoing clinical studies: a phase II study in patients with melanoma (NCT05423262) and a phase II study in patients with gastric cancer (NCT06038578).

The results of this study should be interpreted in light of its limitations. In particular, the small number of enrolled patients and variability in clinicopathological characteristics among patients may have influenced the outcomes.

## Supplementary Information

Below is the link to the electronic supplementary material.

Supplementary file1 (DOCX 637 KB)

## Data Availability

The datasets generated during and/or analyzed during the current study are available from the corresponding author upon reasonable request.
