# Supplementary material for: A phase I study of TRK-950, an Anti-CAPRIN-1 antibody, as monotherapy and in combination with nivolumab in Japanese patients with advanced solid tumors
Source: Invest New Drugs. 2026 Apr 14;44(3):211–25. doi: 10.1007/s10637-026-01609-z (PMC13486075; doi:10.1007/s10637-026-01609-z)
Supplement: Supplementary file 1 [file 10637_2026_1609_MOESM1_ESM.docx]

**Supplementary information**

TRK-950 5 mg/kg weekly (N=3)

Enrolled (N=7)

**Monotherapy (Part 1)**

TRK-950 10 mg/kg weekly (N=4)

TRK-950 10 mg/kg weekly + NIVO (N=3)

Enrolled (N=6)

**Combination with NIVO (Part 2)**

TRK-950 20 mg/kg biweekly + NIVO (N=3)

**Fig. S1 Patient disposition (monotherapy and combination with NIVO)**

Part 1 followed a 3 +3 design with doses ranging from 5–10 mg/kg weekly. Part 2 also followed a 3 + 3 design with TRK-950 doses 10 mg/kg weekly and 20 mg/kg biweekly in combination with nivolumab 240 mg biweekly. Abbreviations: NIVO, nivolumab

10

100

1000

0

24

72

168

336

Time from the first administration in cycle 1 (h)

Serum concentration of TRK-950 (μg/mL)

TRK-950 5 mg/kg weekly

TRK-950 10 mg/kg weekly

TRK-950 10 mg/kg weekly + NIVO

TRK-950 20 mg/kg biweekly + NIVO

**Fig. S2 Time course of serum TRK-950 concentrations**

Serum TRK-950 concentrations were measured over time after the first dose in cycle 1 for patients receiving TRK-950 at 5 mg/kg weekly (N=3), 10 mg/kg weekly (N=4), 10 mg/kg weekly with nivolumab (N=3), and 20 mg/kg biweekly with nivolumab (N=3). Mean serum concentrations with standard deviations are shown.

**Fig. S3 Changes in serum cytokine concentrations after TRK-950 administration**

Serum cytokine concentrations were assessed in patients who received TRK-950 at 5 mg/kg weekly (N=3) or 10 mg/kg weekly (N=4). The cytokines analyzed included IL-6 (interleukin-6), TNF-α (tumor necrosis factor-α), IFN-γ (interferon-γ), IL-1β (interleukin-1β), IL-10 (interleukin-10), and IL-2 (interleukin-2). For patients with cancer of unknown primary who received TRK-950 at 5 mg/kg weekly, predose samples on day 22 were not collected. Values below the lower limit of quantification (LLOQ) were not plotted. IL-2 concentrations were below the LLOQ in all samples.

**Table S1** Summary of the blood sampling schedule for the analysis of TRK-950 and nivolumab concentration

| TRK-950 dosage | Number of patients | Drug | PK sampling schedule |
| --- | --- | --- | --- |
| Part 1 |  |  |  |
| 5 and 10 mg/kg weekly, intravenous administration over 60 min.  Days 1, 8, 15 and 22 for 28-day cycle. | 3 (5 mg/kg) 4 (10 mg/kg) | TRK-950 | Cycle 1:  Day 1; Predose, 5 min, 1, 6, 24 and 72 h after end of infusion  Day 8, 15 and 22; Predose and 5 min after end of infusion  Cycle 2–8:  Day 1, 8, 15 and 22; Predose and 5 min after end of infusion  Cycle ≥ 9:  Day 1; Predose  Follow up visit |
| Part 2 |  |  |  |
| 10 mg/kg weekly, intravenous administration over 60 min.  Days 1, 8, 15 and 22 for 28-day cycle. | 3 | TRK-950 | Cycle 1:  Day 1; Predose, 5 min, 3, 6, 24 and 72 h after end of infusion  Day 8, 15 and 22; Predose and 5 min after end of infusion  Cycle 2–6:  Day 1, 8, 15 and 22; Predose and 5 min after end of infusion  Cycle ≥ 7:  Day 1; Predose  Follow up visit |
|  |  | Nivolumab | Cycle 1 and 3:  Day 1; Predose, 5 min, 3 h after end of infusion  Day 8; During visit (Predose of TRK-950)  Day 15; Predose |
| 20 mg/kg biweekly, intravenous administration over 60 min.  Days 1 and 15 for 28-day cycle. | 3 | TRK-950 | Cycle 1:  Day 1; Predose, 5 min, 3, 6, 24 and 72 h after end of infusion  Day 8; During visit  Day 15; Predose and 5 min after end of infusion  Day 22; During visit  Cycle 2–6:  Day 1 and 15; Predose and 5 min after end of infusion  Cycle ≥ 7:  Day 1; Predose  Follow up visit |
|  |  | Nivolumab | Cycle 1:  Day 1; Predose, 5 min, 3 h after end of infusion  Day 8; During visit  Day 15; Predose  Cycle 3:  Day 1; Predose, 5 min, 3 h after EOI  Day 15; Predose |

**Table S2** Exposure metrics of nivolumab

| Patients (primary tumor) | Cycle 1 | | | Cycle 3 | | |
| --- | --- | --- | --- | --- | --- | --- |
|  | C_max_ (μg/mL) | C_min_ (μg/mL) | C_avg_ (μg/mL) | C_max_ (μg/mL) | C_min_ (μg/mL) | C_avg_ (μg/mL) |
| TRK-950 10 mg/kg weekly + NIVO |  |  |  |  |  |  |
| Pt#8 (Melanoma) | 81.6 | 23.7 | 39.4 | 107 | 67.0 | 84.0 |
| Pt#9 (Esophagus) | 117 | 29.9 | 41.1 | – | – | – |
| Pt#10 (Lung) | 70.6 | 11.7 | 23.5 | – | – | – |
| TRK-950 20 mg/kg biweekly + NIVO |  |  |  |  |  |  |
| Pt#11 (Lung) | 69.4 | 23.9 | 34.5 | – | – | – |
| Pt#12 (Oropharyngeal) | 80.0 | 24.0 | 39.0 | – | – | – |
| Pt#13 (Kidney) | 61.7 | 17.0 | 30.1 | – | – | – |
| Mean ± Standard deviation | 80.1 ± 19.5 | 21.7 ± 6.4 | 34.6 ± 6.8 |  |  |  |

Abbreviations: –: no data; AUC_last_: area under the serum concentration–time curve from time zero to the last quantifiable time point; C_avg_, average serum concentration (AUC_last_ divided by the last quantifiable time point); C_max_: maximum serum concentration; C_min_: trough serum concentration; NIVO: nivolumab; Pt: patient.

**Table S3** Summary of TRK-950 immunogenicity

| Immunogenicity status | Dose level 1 (5 mg/kg), n (%) | Dose level 2 (10 mg/kg), n (%) | TRK-950 (10 mg/kg weekly + NIVO), n (%) | TRK-950 (20 mg/kg biweekly + NIVO), n (%) | All patients, n (%) |
| --- | --- | --- | --- | --- | --- |
| Patients | 3 | 4 | 3 | 3 | 13 |
| Evaluable patients | 3 (100) | 4 (100) | 3 (100) | 3 (100) | 13 (100) |
| Inconclusive patients | 0 | 0 | 0 | 0 | 0 |
| ADA negative patients | 3 (100) | 4 (100) | 2 (66.7) | 3 (100) | 12 (92.3) |
| ADA positive patients |  |  |  |  |  |
| NAb negative | 0 | 0 | 0 | 0 | 0 |
| NAb positive | 0 | 0 | 1 (33.3) | 0 | 1 (7.7) |
| Not evaluable patients | 0 | 0 | 0 | 0 | 0 |

Abbreviations: ADA: anti-drug antibody; Nab: neutralizing antibody; NIVO: nivolumab.

**Table S4** TRK-950 Immunogenicity evaluation per patient

| Patient | Pre Dose | Post Dose 1 | Post Dose 2 | Post Dose 3 | Post Dose 4 | Post Dose 5 | Post Dose 6 | Post Dose 7 | Post Dose 8 | F/U |
| --- | --- | --- | --- | --- | --- | --- | --- | --- | --- | --- |
| TRK-950 5 mg/kg weekly |  |  |  |  |  |  |  |  |  |  |
| Pt#1 (Cancer of unknown primary) | Neg | Neg | Neg | Neg |  |  |  |  |  | Neg |
| Pt#2 (Pancreas) | Neg | Neg | Neg |  |  |  |  |  |  | Neg |
| Pt#3 (Ovary) | Neg | Neg | Neg |  |  |  |  |  |  | Neg |
| TRK-950 10 mg/kg weekly |  |  |  |  |  |  |  |  |  |  |
| Pt#4 (Colorectal cancer) | Neg | Neg | Neg | Neg |  |  |  |  |  | Neg |
| Pt#5 (Pancreas) | Neg | Neg | Neg |  |  |  |  |  |  | Neg |
| Pt#6 (Colorectal cancer) | Neg | Neg |  |  |  |  |  |  |  | Neg |
| Pt#7 (Melanoma) | Neg | Neg | Neg | Neg | Neg | Neg | Neg | Neg | Neg | Neg |
| TRK-950 10 mg/kg weekly + NIVO |  |  |  |  |  |  |  |  |  |  |
| Pt#8 (Melanoma) | Neg | Neg | Neg | Neg | Neg | Neg | Neg |  |  | Neg |
| Pt#9 (Esophagus) | Pos (NAb Pos) | Pos (NAb Neg) | Pos (NAb Pos) |  |  |  |  |  |  | Neg |
| Pt#10 (Lung) | Neg | Neg | Neg |  |  |  |  |  |  | Neg |
| TRK-950 20 mg/kg biweekly + NIVO |  |  |  |  |  |  |  |  |  |  |
| Pt#11 (Lung) | Neg | Neg | Neg |  |  |  |  |  |  | Neg |
| Pt#12 (Oropharyngeal) | Neg | Neg | Neg |  |  |  |  |  |  | Neg |
| Pt#13 (Kidney) | Neg | Neg | Neg |  |  |  |  |  |  | Neg |

Abbreviations: F/U: follow up; NAb Neg: neutralizing antibody-negative; NAb Pos: neutralizing antibody-positive; Neg: anti-drug antibody negative; Pos: anti-drug antibody positive; Pt: patient.
